# Impacts of Sand Burial and Wind Erosion on Regeneration and Growth of a Desert Clonal Shrub

**DOI:** 10.3389/fpls.2018.01696

**Published:** 2018-12-11

**Authors:** Baoli Fan, Changming Zhao, Xiaowei Zhang, Kun Sun

**Affiliations:** ^1^College of Life Science, Northwest Normal University, Lanzhou, China; ^2^State Key Laboratory of Desertification and Aeolian Sand Disaster Combating, Gansu Desert Control Research Institute, Lanzhou, China; ^3^State Key Laboratory of Grassland Agro-Ecosystems, School of Life Sciences, Lanzhou University, Lanzhou, China; ^4^Forestry College, Gansu Agricultural University, Lanzhou, China

**Keywords:** *Calligonum mongolicum*, clonal fragment, clonal integration, physiological and biochemical, sand burial, wind erosion

## Abstract

Sand burial and wind erosion caused by sand movement are common phenomena in desert environments, but the effects on clonal shrub have rarely been investigated. Here, we assessed how sand movements affect the population regeneration capacity of juvenile clonal fragments of the shrub *Calligonum mongolicum* growing in mobile desert sand dunes. We investigated the population status and natural regeneration capacity in three types of mobile dunes (heavy wind erosion, heavy sand burial and moderate sand burial). Clonal propagation of *C. mongolicum* was markedly different across sites. Moderate sand burial sites had the largest ramet density and bud number per unit length of rhizome, and the overwinter survival rate was significantly higher at sand burial sites than at wind erosion sites, suggesting that *C. mongolicum* may have well adapted to the moderate sand burial environment. We further examined the effects of clonal integration on clonal regeneration of this species. Physiological, biochemical and morphological characteristics of parent and daughter ramets growing in heterogeneous sandy habitats (sand burial or wind erosion) were measured. The results showed that being connected or severed from the maternal plant critically determined survival of daughter ramets on wind eroded rhizomes. When eroded rhizomes remained connected, the mother ramets had the highest chlorophyll a, b and a + b contents. However, both the mother plant and the daughter ramets undergoing erosion had higher proline and soluble protein levels than sand buried ramets. Meanwhile, the daughter ramets undergoing sand burial had higher photosynthetic rates (*P*_n_), chlorophyll fluorescence parameters (*F*_m_ and *F*_o_), and phenotypic traits of assimilating shoots, i.e., node number, length and volume than wind-eroded ramets. However, significant differences with mother plants, whether connected or severed, were very limited. It was concluded that moderate sand burial environments promoted clonal reproduction and growth of *C. mongolicum*. Additionally, physiological integration with mother raments in favorable conditions can alleviate stress on daughter ramets exposed to wind erosion. This physiological effect may do not occur for sand buried daughter ramets. These survival strategies and phenotypic responses should be carefully considered in shrub and sand dune management in sand fixation plantations of *C. mongolicum*.

## Introduction

Windblown sand movement is a common phenomenon in deserts (Maun, 1996; Liu et al., 2014). It can either bury vegetation, or conversely, denude the plant and roots through erosion (Xu et al., 2013). Individual plants and plant parts in deserts often experience heterogeneity in sand coverage (Liu et al., 2014). Previous studies have found that there is considerable small-scale spatial variation in the degree of sand movement and the associated degree of burial or denudation of desert plants (Maestre and Reynolds, 2006; Okayasu et al., 2012; Xu et al., 2013). To cope with adverse environments, the majority of indigenous plants in arid regions have evolved different strategies (Su et al., 2009). One of these strategies is to reproduce asexually by means of clonal growth (Maun, 1996). For clonal plants growing in arid dune environments, rare and irregular seedling recruitment is common even when seeds are regularly produced (Eriksson, 1989; Mandujano et al., 2001; Li et al., 2015). This is because frequent sand movement and unpredictable rainfall often lead to failure in seedling recruitment as long-lived perennials often have extended juvenile stages. In contrast, prior to establishment vegetative offspring receive support including water, carbohydrates, and other nutrients from the parent plant at least until it is established (Li et al., 2015). In addition, clonal reproduction permits the effects of deleterious genetic alleles to be masked at heterozygous states, thereby increasing overall fitness per plant (Zhou et al., 2017). Clonal integration has also been shown to enhance plant survival under sand burial (Yu et al., 2001, 2004, 2010), promotes colonization in resource-poor or stressful habitats (Oborny et al., 2000; Song et al., 2013; Xu et al., 2013; Lechuga-Lago et al., 2016). In heterogeneous environments, due to the ability of clonal plants to share parental resources gives them a competitive advantage over non-clonal plants, (Golubski et al., 2008; Oborny et al., 2012; Dickson et al., 2014), thus daughter ramets have a better chance of survival if they remain attached to the parent plant or mother ramet (Balestri and Lardicci, 2013).

Bud count is an important indicator of regeneration potential, and research on clonal and bud bank traits and performances in European and high altitude settings are well described by Klimeš and Klimešová (2000), Pausas and Bradstock (2007), Klimešová and Klimeš (2008) and Klimešová et al. (2011) after fire exposure in Australia. Numerous studies have examined the effects of sand burial on the survival and growth of clonal plant fragments (Dong et al., 2010; Li et al., 2013; Luo and Zhao, 2015a,b). Several studies have also tested the regeneration capacity and subsequent growth of clonal fragments after burial or wind erosion in natural desert environments (Dong et al., 2011; West et al., 2012; Luo and Zhao, 2015b). However, to date, little is known regarding the ability of clonal rhizomatous shrubs to adapt to harsh desert environments characterized by the exposure of clonal plant. Many long-lived shrubs that survive adverse conditions regenerate naturally in mobile sand dunes and these shrubs play a more important role as windbreaks and sand fixation, especially in spring, when sand movement is frequent. Thus, it is important to improve our understanding about the capacity of clonal regeneration of those shrubs that show strong natural regeneration in mobile sand dunes (Huang et al., 2015). Filling these knowledge gaps require field studies that both consider the capacity for clonal regeneration on the population level and assesses the parental effects on the clones themselves.

*Calligonum mongolicum*, a windbreak and sand-fixation pioneer species, occurs naturally in mobile dunes and plays an important role in protecting ecological security in western China. Knowledge of the processes responsible for the natural regeneration of pioneer species during of sand dune stabilization is surprisingly rare (Fan et al., 2018), but is necessary for effective desert control. *C. mongolicum* displays strong clonal regeneration ability in mobile sand dunes, however, available data on the clonal growth pattern of this species are scarce and little is known on the impact of physiological integration between parents and offspring. This study focused on the effects of sand movement on population regeneration and the generative capacity of the bud bank of *C. mongolicum* juvenile shrubs. The study also examined the effects of wind erosion and sand burial on the physiological, biochemical and morphological characteristics of the parental and offspring ramets. To our knowledge, this work is the first to examine clonal regeneration and clonal integration of *C. mongolicum* in a heterogeneous mobile sand dune environment.

## Materials and Methods

### Plant Species and Site Description

*Calligonum mongolicum* is a dominant native perennial shrub in active sand dunes in the arid deserts of northern China (Fan et al., 2018). Well adapted to harsh climate, the foliage of *C. mongolicum* consists of slender, highly branched green to gray–green branchlets that bear small minute scale-leaves. Although *C. mongolicum* populations can propagate sexually and asexually in mobile sand dunes, seedlings appear to suffer high mortality, and therefore clonal reproduction and growth seem to play a major role in the natural regeneration and maintenance of populations in mobile dune habitats. This species is capable of forming several horizontal rhizomes from each node sited along the principal root. The principal roots of *C. mongolicum* are rather short compared to its vertical shoots, i.e., the root length to shoot length ratio is around 0.65 ± 0.08 (mean ± SE), the minimum is 0.39, and the maximum is 0.83. Following sand burial, daughter ramets are formed as new root branches emerge from vegetative buds located at the nodes of buried horizontal roots or shoots.

This study was carried out in mobile sand dunes near the Minqin meteorological station (101°05′E, 38°38′N), in Gansu Province, northwest China. Minqin is adjacent to the Badain Jaran Desert in the northwest and the Tengger Desert to the east. The area has an arid desert climate with an average annual temperature of 7.8°C. Precipitation is usually the only source of water for desert plant growth, and the average annual precipitation is 116.5 mm, with average annual potential evaporation of 2383.7 mm (Fan et al., 2018). The mean wind speed is 2.4 m.s^-1^ and the average number of days with gales (i.e., a wind velocity ≥ 17 m.s^-1^) is 27.4 days per year. The fertility of all soil types in this area is very low due to the harsh climate and sparse desert vegetation.

### Experimental Design

Our research consisted of two sequential field experiments. The first experiment investigated the effects of plot type (comparing heavy wind erosion, heavy sand burial, and moderate sand burial plots) on the population growth and clonal regeneration of *C. mongolicum*. However, almost no horizontal rhizomes were observed in the heavy sand burial microhabitats, thus this treatment (site) was dropped from subsequent investigations. The second experiment assessed survival and effects of clonal integration of *C. mongolicum* in two heterogeneous sand microhabitats (moderate sand burial and heavy wind erosion).

### Experiment 1

#### Vegetation Survey

During early September, 2015, we conducted a vegetation survey in three distinct microhabitats, including the windward sides of dunes (referred to hereafter as ‘heavy wind erosion plots’), plots suffering from heavy sand burial (‘heavy sand burial plots’), and plots that alternated from wind erosion to sand burial (‘moderate sand burial plots’). There were three 20 m × 20 m replicates of each of these microhabitats. We recorded dead shoot percentage of mature mother ramets in each plot and dead shoot percentage were recorded. We assessed the density of mature, seed seedling and clonal juvenile (<30 cm in height) shrubs, mature shrub height and basal diameter of *C. mongolicum* in each of nine 20 m × 20 m plots. Shrub basal diameter and height were measured from where the main root initiated, not at ground level. On heavy wind eroded plots, basal diameter was measured aboveground level, while in heavy and moderate sand burial plots, we excavated the trunk to measure basal diameter at the point where we found an obvious color change, which marked the start of the main root.

#### Horizontal Rhizome Condition

To measure the condition of horizontal rhizomes over time, in early spring 2015, we selected and marked 20 horizontal roots found on plants in the moderate sand burial and wind erosion microhabitats. Horizontal rhizomes existing in heavy sand burial plots were not easily excavated, and no buds emerge from the heavily buried horizontal rhizomes, our observations did not include data from the heavy sand burial plots. Due to the considerable sand movement in some plots over the monitoring period some roots died after they were severed from their mother shrubs. Consequently, we were only able to monitor 11 roots in the erosion plots and 15 roots in the moderate sand burial plots. On all roots, the number of buds and the number of clonal offspring (i.e., ramets) were counted at three points: in the early spring of 2015, in later autumn 2015, and in spring 2016. We then calculated the bud survival percentage and the overwinter survival rate at each plot.

### Experiment 2

#### Clonal Integration

**FIGURE 1:**
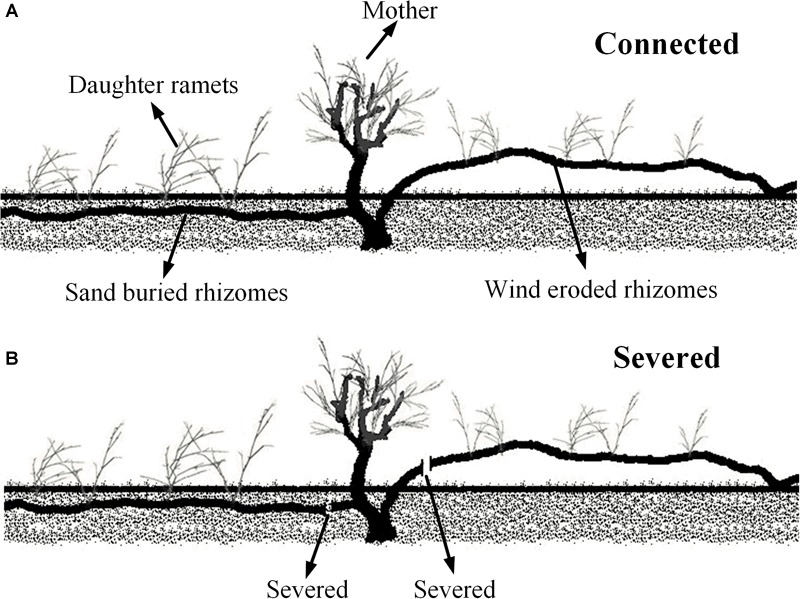
Diagram of Experiment 2. **(A)** Connected treatment; **(B)** severed treatment.

To assess the effects of maternal plant survival on the growth of daughter ramets in different wind and sand environments, we chose fragments that including mother ramets attached with two horizontal rhizomes, of which one rhizome lived in a moderate sand buried microhabitat, and the other one was totally eroded and exposed to the air (Figure 1A). We manually standardized the growing conditions of clonal fragments according to its microhabitat; for instance, we ensured that rhizomes buried in the sand were entirely buried, and that the wind eroded rhizomes were entirely eroded (Figure 1A), each treatment with three replicates. All mother ramets were similar in growth and condition, as were the daughter ramets. The distance between the mother and daughter ramets was between 20 and 40 cm. In late spring 2016, in each plot measurements were taken on all (mother and daughter) ramets. Connections between ramets were then severed (Figure 1B) and the same measurements were retaken 4 weeks later. The data collection included: the morphology of assimilating shoots (i.e., length, diameter, assimilating shoots number per cluster, cluster number per branch and node number of shoots), chlorophyll content concentration, gas exchange parameters and chlorophyll fluorescence. The *C. mongolicum* leaf is a branchlet with reduced leaves (assimilating shoots), therefore the length and diameter of these branchlets were measured with a micrometer. Thus, the volume of assimilating shoots was estimated by: LA = *L*_n_ ×*D^2^× π/4*, where *L*_n_ is the length of assimilating shoots, and *D* is the diameter of assimilating shoots. The physiological and biochemical parameters measured are included in the following section.

#### Physiological and Biochemical Parameters of Assimilating Shoots

The gas exchange parameters of mature assimilating shoots were recorded using a portable open-path gas exchange system with a CO_2_ control (Li-6400, LI-COR Biosciences, Inc., Lincoln, United States). On July 1, 2016, measurements were taken between 10:00 and 12:30 am in full sun on five replicates from each ramet type. The net photosynthetic (*P*_n_) rate, stomatal conductance (Cond), and transpiration (Trmmol) rate were determined for the branchlets of 3 plants of each ramet type under an artificial light source with a photosynthetic photon flux density (PPFD) of 1800 μmol m^-2^ s^-1^ (provided by a Li-6400-02 LED light source) and an ambient concentration of CO_2_ concentration. Assimilating shoots used for photosynthetic measurements were marked and sampled at the end of the experiment, and the surface area of each marked assimilating shoots was determined using a LI-3000A planimeter (LI-COR). Surface area measurements were then used to calculate the net photosynthetic rate per unit area of the assimilating shoots.

Following the gas exchange measurements on each clonal fragment, mature leaves were selected from the south side of the crowns. These were placed in opaque plastic bags, cooled by liquid nitrogen and transported to the laboratory. Proline, total soluble sugars and soluble protein content were determined and measured following the methods of Troll and Lindsley (1955); Spiro (1966), and Bradford (1976), respectively.

#### Chlorophyll Fluorescence

In parallel with the gas exchange parameters, chlorophyll fluorescence was measured by a pulse amplitude modulated portable fluorometer (PAM 2100. Walz, Germany). Assimilating shoots were dark adapted for 30 min, after which the minimal fluorescence level (*F*_o_) was measured by low modulated light and the maximal fluorescence level (*F*_m_) was determined by a saturating pulse on the dark-adapted branchlets. The maximum quantum yield of PSII (*F*_v_/*F*_m_) was then calculated using the equation *F*_v_/*F*_m_ = (*F*_m_ -*F*_o_)/*F*_m_ after (Genty et al., 1989). The steady-state fluorescence (*F*_s_) was recorded after 6 min of light adaptation. Maximal fluorescence level in a light-adapted state (using a saturating pulse, *F*_m_′) and the minimal fluorescence level (using far-red light, *F*_o_′) was determined. The effective quantum yield of PSII (Φ_PSII_), photochemical quenching (qP) and electron transport rate (ETR) were then calculated using the equation: Φ_PSII_ = (*F*_m_′ -*F*_s_)/*F*_m_′; qP = (*F*_m_′ -*F*_s_)/(*F*_m_′ -*F*_o_′); ETR = PAR × 0.5 × Φ_PSII_ × 0.84, after Genty et al., 1989).

#### Statistical Analyses

One-way ANOVA was used to compare the differences in the population of *C. mongolicum*, clonal regeneration features, clonal fragment under different sand environments, leaf morphology and physiological parameters of mother and daughter ramets. Where significant differences were found, multiple comparisons using LSD tests at *P* < 0.05 were performed. Data were tested for homogeneity prior to determining ANOVAs or conducting multiple comparisons. All statistical tests were performed using SPSS 16.0 software. Data means ± SE and figures were calculated using Origin 8.0.

## Results

### Population Features and Clonal Regeneration in Three Mobile Sand Dune Habitats

Population of *C. mongolicum* in eroded, heavily buried and moderate burial sites expressed distinctly different morphologies. At the wind-eroded site, mature shrubs were flattened, the top branches were dead, and many horizontal rhizomes were exposed to the air. At the heavy sand burial sites, only the very top of the exposed shrub survived. The dead shoot percentage at both the eroded and heavily sand buried plots were significantly higher than at the moderate sand burial plot (Table 1).

**Table 1:** Description of populations of *Calligonum mongolicum* under the three sand dune conditions.

Sand dune conditions	Mother ramets status	Percentage of flattened plants	Dead shoot percentage (%)
Heavy wind erosion	100% wind eroded	100%	63.28 ± 7.72^a^
Heavy sand burial	100% sand buried	No	63.76 ± 8.24^a^
Moderate sand burial	100%	No	5.80 ± 2.91^b^

*Different lowercase letters denote significant difference (p < 0.01) among sand dune environments*.

**FIGURE 2:**
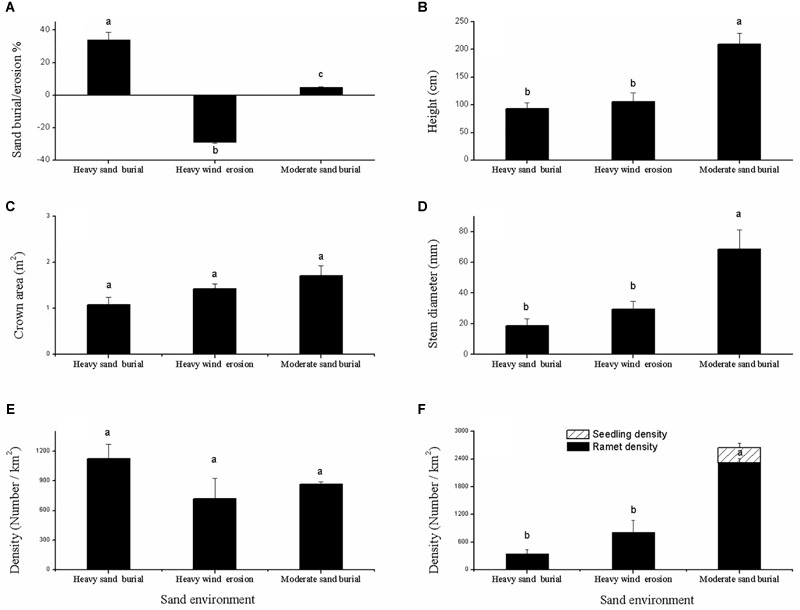
Description of the population dynamics of *Calligonum mongolicum* in various sand dune environments, including heavily buried, moderately buried, and wind-eroded sites. **(A)** Sand buried or wind eroded depth of mature shrub; **(B)** height of mature shrub; **(C)** crown area of mature shrub; **(D)** stem diameter of mature shrub; **(E)** density of mature shrub; **(F)** density of seed seedlings and clonal ramets. [Different lowercase letters denote significant differences (*p* < 0.05) among sand dune environments].

Shrub height and basal diameter were significantly larger at the moderate sand burial site compared to the heavy sand burial site (Figures 2A,B), but no significant changes were found in the crown area or in the mother ramet population density among the three environments (Figures 2C,E). Seedlings from asexual and sexual reproduction both occurred at the moderate sand burial sites, while seed propagation did not occur at the heavy sand burial or wind eroded sites; at these sites we found only clonal regeneration (Figure 2F). The rate of clonal propagation of *C. mongolicum* was markedly different among these three different environments. Ramet density at the moderate sand burial sites was 431% greater than that at eroded sites, and 241% greater than that at the heavy sand burial sites (Figure 2F).

### Effects of Wind Erosion and Sand Burial on Horizontal Rhizome Fragments

*Calligonum mongolicum* on mobile sand dunes in the experimental site had well developed horizontal rhizomes up to several meters in length that gave rise to ramets (Table 2). Length of horizontal rhizomes in eroded sites was much longer than in sand buried sites (Table 2). Although buds were abundant on all rhizomes, the bud number per unit length of rhizome was 2.23 times greater on plants from sand burial plots than from those at eroded sites. In both environments, ramets had very high mortality rates, with only 10% of juvenile ramets surviving at the end of summer. However, the overwinter survival of rate at sand burial sites was 60%, while it was <30% at eroded sites (Table 2). Moreover, the number of assimilating shoots per unit branch under sand burial was significantly larger than that at eroded sites.

**Table 2:** Clonal growth of horizontal rhizomes and bud banks in natural sand burial and wind erosion environments.

Parameters	Status of horizontal rhizomes		F	*P*
	Sand buried	Wind eroded		
Length of horizontal rhizome (cm) (Minimum ~ Maximum)	32 ~ 370	80 ~ 713	1.33	0.26
Diameter of horizontal root (mm) (Minimum ~ Maximum)	13.7 ~ 23.9	7.9 ~ 17.6	20.85	<0.001
Bud number per unit length of rhizome	1.68 ± 0.21	0.52 ± 0.08	21.64	<0.001
Bud survival percentage (%)	10.88 ± 2.36	10.18 ± 2.9	0.035	0.85
Overwinter survival rates (%)	58.63 ± 5.17	28.44 ± 4.38	15.63	0.001
Number of assimilating shoots per unit branch	0.18 ± 0.04	0.05 ± 0.02	7.24	0.013

### Clonal Fragments in Different Sand Environments

Clonal ramets that sprouted from sand-buried and wind-eroded horizontal rhizomes had significantly different clonal growth characters. Ramet density was much greater from sand-buried than from wind-eroded rhizomes (*P* < 0.05), although spacer length showed a contrary result (*P* < 0.05) (Table 3).

**Table 3:** Description of mother and daughter ramets of *C. mongolicum* in different sand burial and wind erosion environments.

	Mother ramet	Sand-buried rhizome	Wind-eroded rhizome	*F*(*P*)
Burial depth (cm)	13 ~ 21	7.8 ~ 13	/	/
Wind erosion (cm)	/	Nd	8 ~ 28	/
Ramets density (number per unit length)	/	0.085 ± 0.007	0.031 ± 0.005	38.11^∗^
Ramet spacing length (cm)	/	11.92 ± 1.94	33.82 ± 5.59	14.76^∗^

*^∗^Means sig < 0.05; Nd, not determined due to high mortality rates*.

**FIGURE 3:**
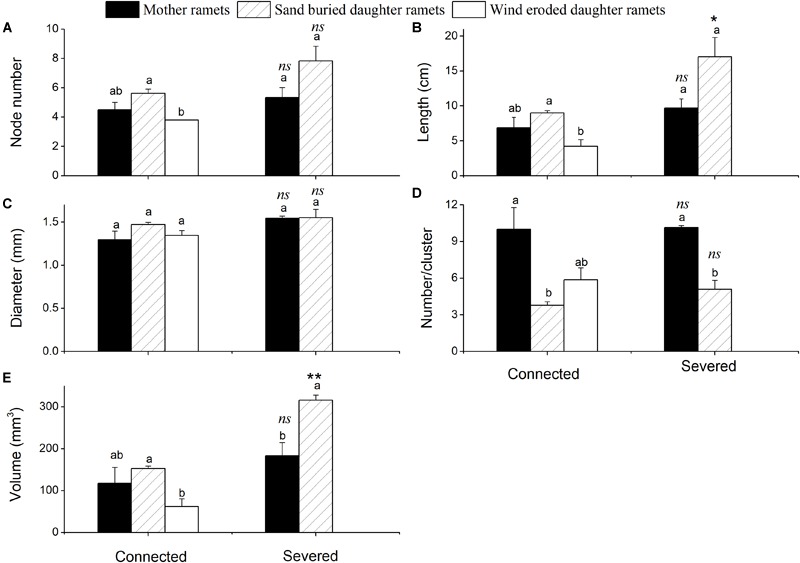
Phenotypic changes of assimilative branches of mother and daughter ramets in *C. mongolicum* at wind eroded and sand accumulated environments under different treatments (mean ± SE). **(A)** Assimilating shoots node number; **(B)** assimilating shoots length; **(C)** assimilating shoot diameter; **(D)** assimilating shoot number per cluster; **(E)** assimilating shoot volume. [Different lowercase letters denote significant differences (*p* < 0.05) among ramets before or after being severed, ^∗^ denotes a significant difference (*p* < 0.05) between the same type of ramets before and after being severed]; ^∗∗^ denotes a significant difference (*p* < 0.01) between the same type of ramets before and after being severed; ns denotes *p* > 0.05].

Sand movement significantly affected the number of assimilating shoot nodes, the length of assimilating shoots and the volume of assimilating shoots of daughter ramets when connected with mother ramets. Each of these parameters was less in wind eroded conditions, although they were not significantly different from mother ramets (Figure 2). Being connected or severed critically determined the survival of daughter ramets at wind-eroded rhizomes. Severing rhizomes at wind-eroded sites caused the total senescence of daughter ramets within 1 week, thus data on these ramets was not available. In contrast, severing rhizomes did not affect the survival of daughter ramets at sand burial sites. However, at sand burial sites the length of assimilating shoots length (*F* = 14.334, *P* < 0.05) and the volume of assimilating shoots (*F* = 190.86, *P* < 0.001) were greater than these in mother ramets following severing. The difference in diameter of assimilating shoots of *C. mongolicum* for all surviving mother and daughter ramets did not significantly differ, while the number of assimilating shoots per cluster in mother ramets was larger than in the sand buried daughter ramets, no matter whether they were connected or severed (*F* = 69.99, *P* = 0.004) (Figure 3).

**FIGURE 4:**
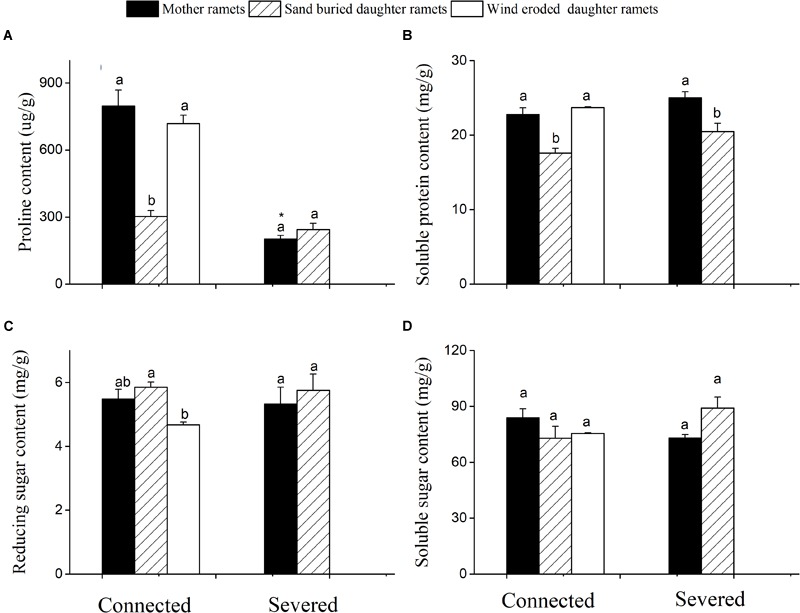
Changes in the biochemical characteristics of *C. mongolicum* mother and daughter ramets when severed or connected under wind eroded and sand burial environments. **(A)** Proline content; **(B)** Soluble protein content; **(C)** Reducing sugar content; **(D)** Soluble sugar content [Different lowercase letters denote significant differences (*p* < 0.05) among ramets before or after being severed, ^∗^ denotes significant difference (*p* < 0.05) between the same type of ramets before and after being severed].

When rhizomes remained connected, both mother plant and the daughter ramets under eroded conditions had greater proline and soluble protein content than sand buried ramets (Figures 4A,B). However, when rhizomes were severed the proline content of mother ramets significantly declined from 705 to 245 μg/g (Figure 4A). Severed or connected, proline and protein content at sand burial sites did not significantly differ. Meanwhile, reducing and soluble sugars did not differ between severed and connected rhizome treatments or among clonal fragments before or after being severed with the exception of reducing sugar content between mother and wind eroded daughter ramets (Figures 4C,D).

### Net Photosynthetic Rate and Chlorophyll Content

**FIGURE 5:**
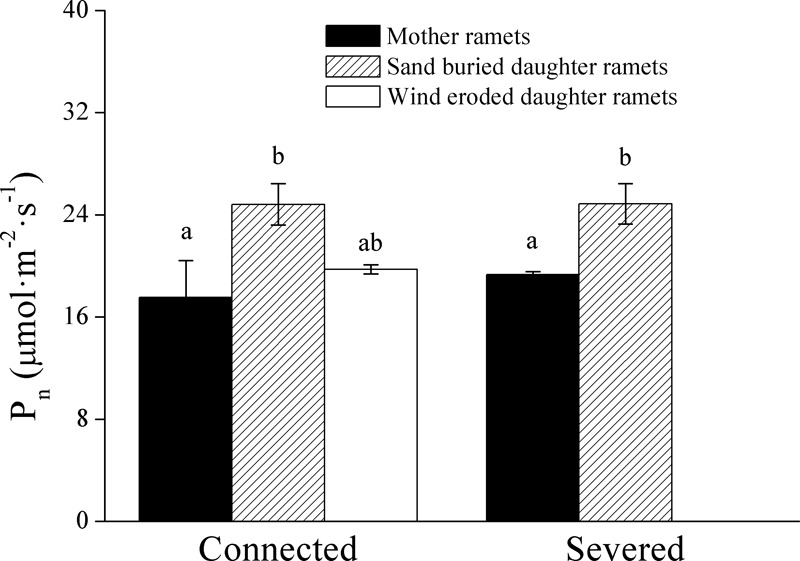
The effect on net photosynthetic rate (*P*_n_) of severing the connection between *C. mongolicum* mother (M) and daughter (R) ramets when severed or connected under eroded (Rw) or sand burial (Rs) environments. [Different lowercase letters denote significant differences (*p* < 0.05) among clonal fragments before or after being severed.

When ramets remained connected, *P*_n_ values in both the mother and daughter ramets were greater at sand burial treatments than at wind-eroded sites (Figure 5). After rhizomes were severed *P*_n_ values were greater in sand burial daughter ramets than mother ramets (Figure 5).

**FIGURE 6:**
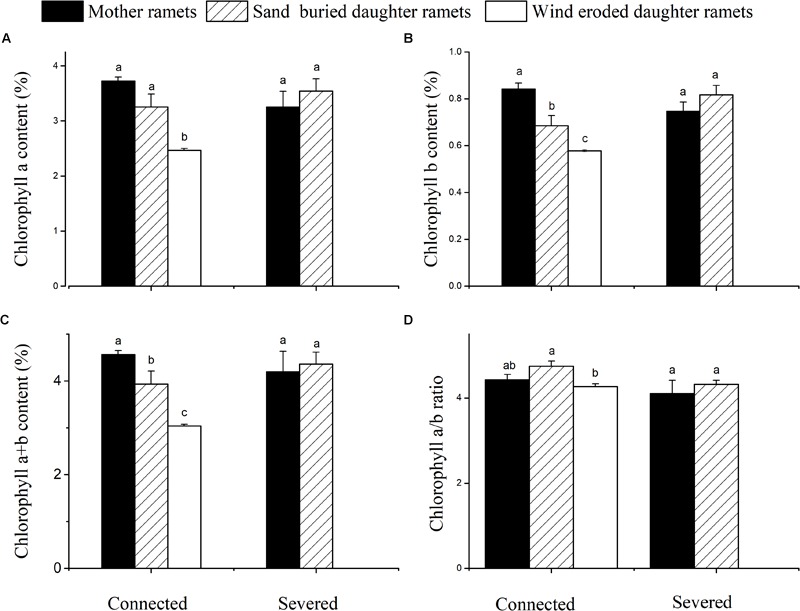
Chlorophyll content changes in mother and daughter ramets when connected and severed in *C. mongolicum* growing in wind eroded and sand accumulated environments. **(A)** chlorophyll a; **(B)** chlorophyll b; **(C)** chlorophyll a + b; **(D)** chlorophyll a/b. [Different lowercase letters denote significant differences (*p* < 0.05) among clonal fragments before or after being severed.

Chlorophyll a, b and total chlorophyll contents of mother ramets were higher at sand-buried sites before severing, while ramets at wind-eroded sites had the least chlorophyll a, b and a + b contents. However, no significant differences in chlorophyll content between ramets in either connection condition were apparent (Figure 6).

### Chlorophyll Fluorescence Parameters

The potential quantum efficiency of PSII (*F*_v_/*F*_m_) did not differ among the three types of ramets and was unaffected when the rhizomes were severed. When rhizomes remained intact, *F*_m_ and *F*_o_ were considerable greater in sand buried ramets than in mother or wind eroded ramets (*P* < 0.001). In addition, there were no significant difference between mother and wind-eroded ramets (*P* > 0.05). Moreover, *F*_m_ and *F*_o_ values did not significantly differ between mother and sand-buried ramets after rhizomes were severed (*P* > 0.05). However, the *F*_m_ and *F*_o_ values in the mother ramets themselves were significantly different after rhizomes were severed (both *P* < 0.05) (Table 4).

**Table 4:** Chlorophyll fluorescence parameters in assimilating shoots of different types of ramets under connected and severed conditions.

		Mother ramet	Sand buried ramet	Wind eroded ramet
*F*_m_	Connected	1.42 ± 0.05bA	2.16 ± 0.27aA	1.27 ± 0.17b
	Severed	2.04 ± 0.22aB	1.92 ± 0.22aA	-
*F*_o_	Connected	0.34 ± 0.01bA	0.47 ± 0.06aA	0.29 ± 0.04b
	Severed	0.46 ± 0.04aB	0.44 ± 0.05aA	-
*F*_v_/*F*_m_	Connected	0.76 ± 0.01aA	0.78 ± 0.01aA	0.78 ± 0.01a
	Severed	0.77 ± 0.01aA	0.77 ± 0.01aA	-
ΦPSII	Connected	0.59 ± 0.01aA	0.50 ± 0.07abA	0.43 ± 0.03b
	Severed	0.56 ± 0.01aA	0.54 ± 0.01aA	-
qP	Connected	0.86 ± 0.01aA	0.74 ± 0.08abA	0.65 ± 0.04b
	Severed	0.82 ± 0.02aA	0.81 ± 0.01aA	-
ETR	Connected	25.15 ± 0.29aA	21.26 ± 2.94abA	18.16 ± 1.85b
	Severed	24.17 ± 0.43aA	23.07 ± 0.42aA	-

*Different lowercase letters denote significant difference (p < 0.05) among clonal fragments before or after being severed; different uppercase letters denote significant differences (p < 0.05) between the same type of ramets before and after being severed*.

The effective quantum yield of PSII (Φ_PSII_), photochemical quenching (qP) and electron transport rate (ETR) values for the three types of ramets remained constant between the connected and severed conditions. Under connected conditions, Φ_PSII_, qP, and ETR were greatest in mother ramets and not significantly different in sand-buried ramets, but were significantly different for wind eroded ramets (*P* < 0.001). Under severed conditions, these three indicators were not significantly different between mother and sand buried ramets (Table 4).

## Discussion

The shrub *C. mongolicum* is well adapted to desert environments where populations experiencing fast expansion mainly by clonal regeneration and clonal growth (Fan et al., 2018). However, for continual population recruitment, increased colonization capacity depends on juvenile ramet survival, especially when occupying stressful (wind eroded) environments.

### Effects of Sand Movement on the Population Status and Capacity of Clonal Regeneration

Both heavy sand burial and heavy wind erosion can greatly impair the growth and physiology of a plant population (Yu et al., 2008; Lechuga-Lago et al., 2016). Plants growing on the windward side of dunes commonly lose water from their root system due to root exposure by wind erosion (Yu et al., 2008). In addition, plants on the leeward side of the dune are prone to being buried by sand (Li et al., 2013). Only moderate sand burial formed by the interaction of wind erosion and sand burial provides an ideal microhabitat, where plants can propagate (by ramets) quickly and without inhibition. In addition, we found that the proportion of dead shoots at both wind-eroded and sand-buried sites were significantly higher than at the moderately sand-buried plot. This indicates that this was the most favorable environment for growth of *C. mongolicum* shrubs (Table 1). Clonal regeneration also differed among the three different mobile sand environments. In the severe sand burial and wind erosion sites, many fewer daughter ramets were found than at the moderate sand burial site. Moreover, new ramets initialized on rhizomes failed to emerge through the physical barrier of deep sand burial sites and thus increased mortality, as reserves stored in the plant organs were depleted (Yu et al., 2001, 2004). Ramets on rhizomes exposed to serious wind erosion suffered extreme senescence from the lack of moisture (Luo and Zhao, 2015b). Our results demonstrated that the effects of sand burial and wind erosion on clonal plants may have significant effects on ecological succession patterns over several years, as was reported by Mandujano et al. (2007).

Horizontal rhizomes play a critical ecological and physiological role for *C. mongolicum*. They are important clonal organs that produce and maintain belowground buds, which are capable of forming daughter ramets from each bud site on the roots (rhizomes) (Table 2), and provide a vital connection with the mother plant and the ramets, thereby significantly contributing to colonization and dispersal. However, sand movement causes heterogeneous habitats for horizontal rhizomes and hence variance in their survival and function. We found that horizontal rhizomes can extend several meters from the mother ramet, and that longer roots occur at wind-eroded sites than at sand-buried sites because natural wind erosion effectively exposes horizontal rhizomes. On the other hand, we also found that when exposed to the air bud bank density and overall survival rates were much lower at wind-eroded sites than at sand burial sites, where such exposure does not occur (Table 2). This difference in survival likely happens because wind erosion denudes roots, thereby modifying the bud bank size (i.e., the number of buds) and the overwinter survival rate. Because survival and bud production were both higher at sand burial sites, we concluded that moderate sand burial environments promote clonal reproduction to a greater degree than exposure from wind erosion (Table 2). Our results are aligned with the findings of previous studies that indicated that moderate sand burial maintains a moist environment around clonal fragments and protects them from drying out (Maun, 1996; Luo and Zhao, 2015a).

### Adaptive Strategies to Mobile Sand Dune Environments

Though sand movement significantly affects the clonal regeneration, *C. mongolicum* takes some strategies to adapt to mobile sand dune environments. First, the extensive root architecture reflects the plant’s adaptive ability to make best use of unevenly distributed soil resources (Fitter and Stickland, 1991). In our study the root length (vertical principal root) to shoot length ratio found in mature natural populations was lower than 1.0. While each mother ramet of *C. mongolicum* can often include several horizontal rhizomes, and each can extend beyond several meters from the mother ramets, indicating that *C. mongolicum* allocates few resources to principal vertical root tissues, with increasing resource allocation to horizontal roots. This was previously documented for two other rhizomatous dune species, *Ammophila breviligulata* (Maun, 1984) and *Sporobolus virginicus* (Balestri and Lardicci, 2013). The spread of horizontal rhizomes substituting for vertical roots may be an important adaptive strategy used by clonal plants to colonize harsh mobile sand dune habitats (Pitelka and Ashmun, 1985; de Kroon and Hutchings, 1995). This adaptive strategy may also increase plant survival after heavy burial or wind erosion, and/or may permit *C. mongolicum* to forage for water in less affected parts of the dune. This foraging strategy was identified in the clonal plant *Hedysarum laeve* (Li et al., 2015) and a similar strategy may be used by the woodland strawberry, *Fragaria vesca* (Waters and Watson, 2015).

Second, we found a proliferation of ramets on rhizomes that had been buried by sand in the early growing season from 0 ~ 25 cm depth. However, most ramets initiated from these buds died within 1 month of emergence. The high mortality rate of juvenile ramets occurred in the early growing season in both sand environments, with only 10% remaining at the end of the growing season (Table 2). Thus, *C. mongolicum* demonstrated a high ramet turnover in its early life stage, in both mortality and initiation, which could be attributed to the continual windy and drying conditions in the Minqin region. This result is consistent within plants of the same genus (*C. arborescens*) under severe sand burial depths (Luo and Zhao, 2015a). In a moderate sand burial environment, survival depends mainly on plant density as water and nutrient in the Minqin dune environment are quite poor, thus the competition between ramets is severe, and fast ramet turn over may assist in avoiding localized water and nutrient depletion (Dong and Alaten, 1999; Li et al., 2015). This could also be described as an adaptive strategy used by *C. mongolicum* to cope with the highly variable sand environment in this region.

In summary, *C. mongolicum* rhizomes can extend considerable distances and have a high capacity for ramet regeneration, however, this capacity is highly dependent on sand burial state. A habitat with alternating wind erosion and sand burial, i.e., moderate sand burial environment is ideal for clonal reproduction and colonization. In a harsh desert sand environment, we also find that *C. mongolicum* exhibit an exploratory foraging strategy, similar to those found in other clonal plants.

### Effects of Physiological Integration on Clonal Growth in Different Sand Environments

Despite the adaptive strategies of *C. mongolicum*, wind erosion remains a major stress factor in the Minqin region. Results from this study demonstrate that wind erosion led to a reduction in leaf attributes on both mother and daughter ramets. Even so, ramets at wind eroded sites that were unfavorable for growth still survived if they remained connected to the parental plant via the rhizome. Physical connections between ramets transports resources within clonal plants, this integration significantly ensured young ramet survival and permitted continuing development in the harsh wind-blown environment. These findings are consistent with those of Hartnett and Bazzaz (1983); Salzman and Parker (1985), and Roiloa and Hutchings (2012), who demonstrated that resource transport occurs from ramets under favorable conditions to developing ramets in unfavorable sites.

In contrast, sand buried daughter ramets had improved assimilating shoot elongation. This appeared very important for daughter clonal fragment of *C. mongolicum* compared to mother ramets, which increased the number of assimilating shoots per cluster (branchlets), presumably to enhance photosynthesis. Even when rhizomes were severed, daughter ramets maintained growth status as with the mother ramets. The reason for this was explained by Yu et al. (2004); Dong et al. (2010, 2011); and Balestri and Lardicci (2013), who noted that the internodes of clonal plants contain storage materials that can be remobilized by ramets when necessary. Thus, under moderate sand burial conditions, *C. mongolicum* rhizomes and their nodes could also mobilize water and nutrients to the ramets after being disconnected from the mother ramet. Moreover, daughter ramets survived by carrying out photosynthesis, and were able to absorb enough photosynthate for normal growth. Physiological integration through rhizome connection increased the colonization capacity and ramet survival of *C. mongolicum* ramets occupying wind eroded environments.

Sand-buried ramets produced more chlorophyll a, chlorophyll b and total chlorophyll contents than wind eroded ramets when connected. As stated previously these ramets also have a higher photosynthetic capacity. When connected, if daughter ramets suffered from wind erosion, mother ramets showed the same trend as they mobilize resources to reduce plant cell membrane damage by increasing proline and soluble protein content (Luo et al., 2014; Ye et al., 2016). Our results demonstrated that at wind-eroded sites mother and (stressed) daughter ramets had higher proline and soluble protein content than sand buried (i.e., unstressed) ramets did. In parallel with this finding, the photosynthetic rate (*P*_n_) and chlorophyll fluorescence parameters (*F*_m_, *F*_o_, Φ_PSII_, qP, and ETR) showed similar trends, much greater values were recorded from sand buried ramets than from wind eroded ramets under connected conditions. Once daughter ramets were disconnected, *P*_n_ values were similar in mother and sand-buried daughter ramets, showing that sand buried ramets had more stable photosynthetic systems. Ashraf and Harris (2013) proved that moderately sand buried conditions promoted the clonal growth of *C. mongolicum*. However, once daughter ramets were disconnected, proline content, *F*_o_ and *F*_m_ in mother ramets significantly declined to levels found in buried ramets. Taken together, these results demonstrated that mother ramets in favorable conditions could experience stress when connected daughter ramets come under stress. Mother ramets then coordinate a physiological response to the stress. Moreover, this response does not occur for daughter ramets that occupy favorable sites.

## Conclusion

The study focused on the effect of sand movement on population regeneration capacity in *C. mongolicum*. This shrub is uniquely adapted to shifting sand environments in deserts. However, despite its best efforts to mobilize plant internal resources, normally buried plant parts when exposed to the air-causes high mortality. Wind erosion and sand burial were both found to affect physiological, biochemical, and morphological characteristics of both parent and clonal ramets. The study concluded that this plant has a number of strategies to mobilize and coordinate resources to maintain colonization though its clonal rhizomes. In the variable conditions of arid sandy deserts, daughter ramets benefit from clonal integration with the parent ramets, especially under wind eroded and unfavorable environments, which should be carefully considered in shrub and sand dune management of sand fixation plantations of *C. mongolicum*. However, to fully understand the transmission capacity of water, nutrients and energy between *C. mongolicum* clonal ramets, and apply it to plantation management, further research under sand burial and wind erosion conditions is essential.

## Author Contributions

BF conceived and conducted the field study, analyzed the data, and wrote the manuscript. CZ supervised the manuscript. XZ analyzed part of the data. KS supervised and reviewed the manuscript. All authors contributed critically to the drafts and provided approval of the final version of the manuscript for publication.

## Conflict of Interest Statement

The authors declare that the research was conducted in the absence of any commercial or financial relationships that could be construed as a potential conflict of interest.
